# Crystal structure of *cis*-aqua­chlorido­(*rac*-5,5,7,12,12,14-hexa­methyl-1,4,8,11-tetra­aza­cyclo­tetra­decane-κ^4^ *N*)chromium(III) tetra­chlorido­zincate trihydrate from synchrotron data

**DOI:** 10.1107/S2056989015015212

**Published:** 2015-08-22

**Authors:** Dohyun Moon, Jong-Ha Choi

**Affiliations:** a Pohang Accelerator Laboratory, POSTECH, Pohang 37673, Republic of Korea; b Department of Chemistry, Andong National University, Andong 36729, Republic of Korea

**Keywords:** crystal structure, synchrotron radiation, macrocyclic chromium(III) complex, chlorido ligand, aqua ligand, *cis*-geometry, hydrogen bonding

## Abstract

The structure of the title compound, *cis*-[CrCl(cyc*b*)(H_2_O)][ZnCl_4_]·3H_2_O (cyc*b* is *rac*-5,5,7,12,12,14-hexa­methyl-1,4,8,11-tetra­aza­cyclo­tetra­decane; C_16_H_36_N_4_), has been determined from synchrotron data. In the complex cation, the Cr^III^ ion is bound by four N atoms from the tetra­dentate cyc*b* ligand, a chloride ion and one water mol­ecule in a *cis* arrangement, displaying a distorted octa­hedral coordination geometry. The distorted tetra­hedral [ZnCl_4_]^2−^ anion and three additional water mol­ecules remain outside the coordination sphere. The Cr—N(cyc*b*) bond lengths are in the range of 2.0837 (14) to 2.1399 (12) Å while the Cr—Cl and Cr—(OH_2_) bond lengths are 2.2940 (8) and 2.0082 (13) Å, respectively. The crystal packing is stabilized by hydrogen-bonding inter­actions between the N—H groups of the macrocyclic ligand, the O—H groups of the water mol­ecules and the Cl atoms of the tetra­chlorido­zincate anion, leading to the formation of a three-dimensional network.

## Abstract 2

The Cr^III^ ion in the title cationic complex is coordinated by four N atoms from the macrocyclic ligand, one water mol­ecule and one chloride in a *cis* geometry, displaying a distorted octa­hedral environment. The crystal packing is stabilized by N—H⋯Cl, O—H⋯Cl and O—H⋯O hydrogen bonds.

## Chemical context   

Chromium(III) complexes containing *C*-*meso* or *racemic*-5,5,7,12,12,14-hexa­methyl-1,4,8,11-tetra­aza­cyclo­tetra­decane (cyc*a* and cyc*b*) ligands are known to exist in *trans* or *cis* octa­hedral coordination geometries when combined with two auxiliary ligands (House *et al.*, 1983 ▸; Eriksen & Mønsted, 1983 ▸). The cyc*b* ligand readily folds to form the *cis* isomer while the cyc*a* ligand only folds with difficulty into the *trans* isomer. There are five conformational *trans* isomers for the cyclam moiety which differ in the chirality of the *sec*-NH group (Choi, 2009 ▸). Ligands with *trans*-I, *trans*-II or *trans*-V configurations can fold into *cis*-I, *cis*-II and *cis*-V isomers, respectively (Subhan *et al.*, 2011 ▸). Infrared and electronic absorption spectral properties are useful in determining the geometric isomers of Cr^III^ complexes with mixed ligands (Choi *et al.*, 2004 ▸; Choi & Moon, 2014 ▸; Moon & Choi, 2015 ▸). However, it should be noted that the geometric assignments based on spectroscopic studies alone are less conclusive. In order to study the mol­ecular structure and crystal packing mode of a complex containing Cr^III^, the cyc*b* ligand and a ZnCl_4_^2−^ counter-anion, we report herein on the preparation and crystal structure of *cis*-[CrCl(cyc*b*)(OH_2_)]ZnCl_4_·3H_2_O, (I).

## Structural commentary   

In the mol­ecular structure of the complex cation, there is one chlorine atom and one water mol­ecule coordinating the Cr^III^ ion with an O1*A*—Cr1*A*—Cl1*A* bond angle of 85.74 (4)°. The rest of the coordination sites are occupied by four nitro­gen atoms of the tetra­dentate macrocyclic cyc*b* ligand, giving rise to a distorted octa­hedral coordination sphere.
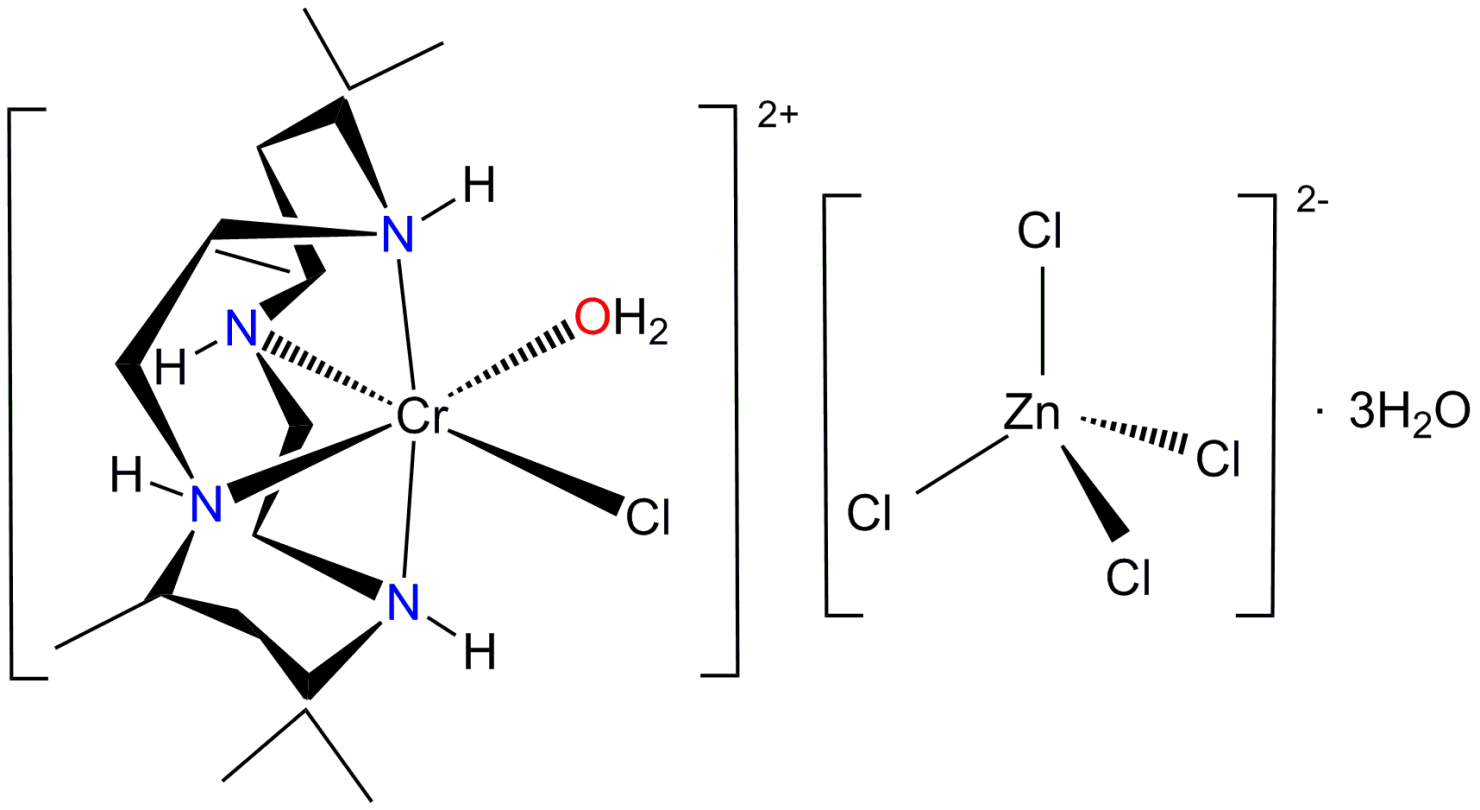


**Figure 1:**
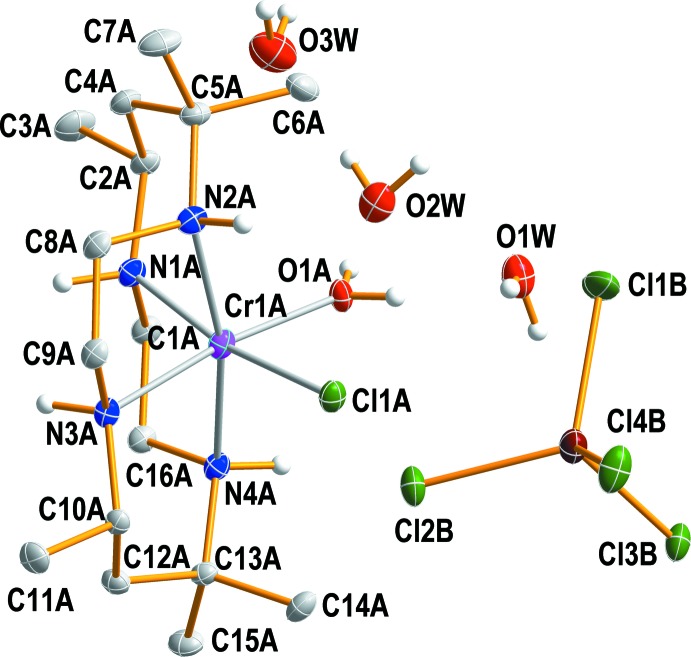
The structure of the mol­ecular entities in compound (I), with displacement ellipsoids drawn at the 30% probability level. H atoms bonded to C atoms have been omitted for clarity.

The cyc*b* ligand is folded about the N2*A*—Cr1*A*—N4*A* line and is in its most stable *cis*-V conformation (Fig. 1 ▸). The Cr—N(cyc*b*) bond lengths are in the range 2.0837 (14) to 2.1399 (12) Å, in good agreement with those observed in *cis*-[Cr(OH)_2_(cyc*b*)]ClO_4_·2H_2_O [2.140–2.142 Å; Bang & Mønsted, 1984 ▸], *cis*-[Cr(NCS)_2_(cyc*b*)]ClO_4_·H_2_O [2.103 (4)–2.147 (4) Å; Byun *et al.*, 2005 ▸], *cis*-[Cr(O_2_CO)(cyc*b*)]Br·H_2_O [2.093 (3)–2.115 (3) Å; Dobrzańska, 2005 ▸], *cis*-[Cr(CN)_2_(cyc*b*)]Cl [2.119 (3)–2.135 (2) Å; Lessard *et al.*, 1992 ▸], or *cis*-[Cr(acac)(cyc*b*)]ClO_4_·0.5H_2_O [acac is acetyl­acetonate; 2.107 (3)–2.133 (3) Å; Byun & Han, 2005 ▸]. The Cr—Cl and Cr—(OH_2_) bond lengths are 2.2940 (8) and 2.0082 (13) Å, respectively. The Cr—Cl bond is slightly shorter than in *trans*-[CrCl(cyc*a*)(OH_2_)](NO_3_)_2_ [2.307 (2) Å; Temple *et al.*, 1984 ▸] or *trans*-[CrCl_2_(Me_2_tn)_2_]Cl [Me_2_tn = 2,2-di­methyl­propane-1,3-di­amine; 2.3253 (7); Choi *et al.*, 2007 ▸]. The length of the Cr—(OH_2_) bond in the title compound is comparable to the values of 2.090 (6) and 1.996 (4) Å found in *trans*–[CrCl(cyc*a*)(OH_2_)](NO_3_)_2_ (Temple *et al.*, 1984 ▸) and *trans*-[CrF(3,2,3-tet)(OH_2_)](ClO_4_)_2_·H_2_O (3,2,3-tet = 1,5,8,12-tetra­aza­undecane; Choi & Lee, 2008 ▸), respectively. The Cl1*A*—Cr1*A*—N1 and O1*A*—Cr1*A*—N3*A* angles are 170.35 (3) and 172.43 (5)°, respectively. The angles N1*A*—Cr1*A*—N2*A* and N3*A*—Cr1*A*—N4*A* are 87.01 (5) and 87.77 (5)°, reflecting the distorted octa­hedral coordination sphere. The tetra­hedral [ZnCl_4_]^2−^ anion and three additional water mol­ecules remain outside the coordination sphere of Cr^III^. The complex anion is distorted due to its involvement in hydrogen-bonding inter­actions. Zn—Cl bonds in the anion span a range from 2.2569 (7) to 2.3131 (8) Å, and the Cl—Zn—Cl angles from 106.02 (4) to 111.49 (3)°.

## Supra­molecular features   

**Figure 2:**
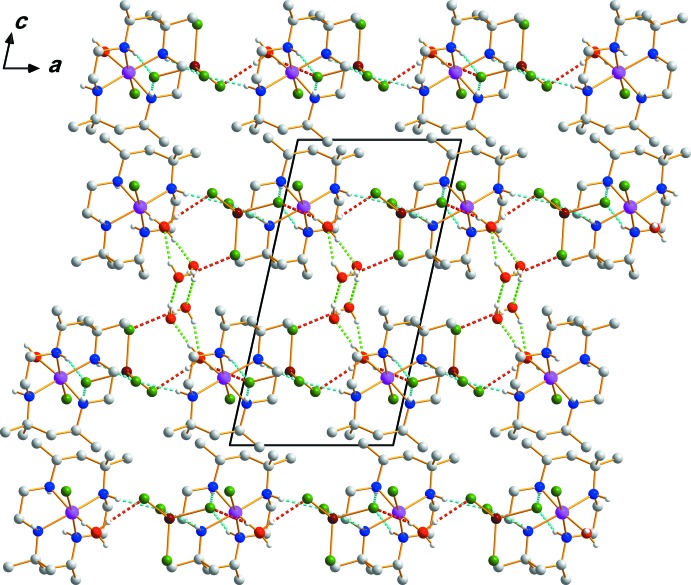
The crystal packing of compound (I), viewed perpendicular to the *ac* plane. Dashed lines represent hydrogen-bonding inter­actions of the types O—H⋯O (light green), O—H⋯Cl (red) and N—H⋯Cl (cyan). H atoms bonded to C atoms have been omitted for clarity.

Extensive hydrogen-bonding inter­actions occur in the crystal structure (Table 1 ▸). The supra­molecular architecture involves hydrogen-bonding inter­actions including the N—H groups of the macrocycles, the O—H groups of coordinating and lattice water mol­ecules as donors, and the anion Cl atoms and O atoms of coordinating and lattice water mol­ecules as acceptors, giving rise to a three-dimensional network structure (Fig. 2 ▸).

## Database survey   

A search of the Cambridge Structural Database (Version 5.36, last update February 2015; Groom & Allen, 2014 ▸) gave 13 hits for Cr^III^ complexes involving the macrocyclic *rac*-5,5,7,12,12,14-hexa­methyl-1,4,8,11-tetra­aza­cyclo­tetra­decane ligand. The crystal structures of *cis*-[Cr(OH)_2_(cyc*b*)]ClO_4_·2H_2_O (Bang & Mønsted, 1984 ▸), *cis*-[Cr(NCS)_2_(cyc*b*)]ClO_4_·H_2_O (Byun *et al.*, 2005 ▸), *cis*-[Cr(O_2_CO)(cyc*b*)]Br·H_2_O (Dobrzanska, 2005 ▸) *cis*-[Cr(CN)_2_(cyc*b*)]Cl (Lessard *et al.*, 1992 ▸), *cis*-[Cr(acac)(cyc*b*)]ClO_4_·0.5H_2_O (Byun & Han, 2005 ▸), *trans*–[CrCl(cyc*a*)(OH_2_)](NO_3_)_2_ (Temple *et al.*, 1984 ▸) and *trans*-[Cr(OH)(cyc*a*)(OH_2_)](ClO_4_)_2_·H_2_O (Goodson *et al.*, 2001 ▸) have been reported previously. However, no crystal structure of the [CrCl(cyc*b*)(OH_2_)]^2+^ cationic complex with any anion was found, although the preparation of *cis*-[CrCl(cyc*b*)(OH_2_)](ClO_4_)_2_·0.4HClO_4_·3H_2_O has been reported (Eriksen & Mønsted, 1983 ▸).

## Synthesis and crystallization   

All chemicals were reagent grade materials and used without further purification. The starting material, *cis*-[CrCl_2_(cyc*b*)]Cl·H_2_O was prepared according to literature procedures (Eriksen & Mønsted, 1983 ▸). Crude *cis*-[CrCl_2_(cyc*b*)]Cl·H_2_O (0.07 g) was dissolved in 4 mL of 0.01 *M* HCl at 353 K and the 1 mL of 6 *M* HCl containing 0.15 g of solid ZnCl_2_ were added to this solution. The mixture was refluxed for 30 min and then cooled to room temperature. The resulting solution was filtered and the filtrate was allowed to stand at room temperature for one day to afford purple crystals of compound (I) suitable for X-ray structural analysis.

## Refinement   

Crystal data, data collection and structure refinement details are summarized in Table 2 ▸. H atoms were placed in geometrically idealized positions and constrained to ride on their parent atoms, with C—H = 0.96–0.98 Å and N—H = 0.98 Å, and with *U*_iso_(H) values of 1.2 or 1.5 × *U*_eq_ of the parent atoms. The hydrogen atoms of water mol­ecules were located in difference maps restrained with O—H = 0.84 Å using DFIX and DANG commands.

## Supplementary Material

Crystal structure: contains datablock(s) I. DOI: 10.1107/S2056989015015212/wm5196sup1.cif

Structure factors: contains datablock(s) I. DOI: 10.1107/S2056989015015212/wm5196Isup2.hkl

CCDC reference: 1419197

Additional supporting information:  crystallographic information; 3D view; checkCIF report

## Figures and Tables

**Table 1:** Hydrogen-bond geometry (, )

*D*H*A*	*D*H	H*A*	*D* *A*	*D*H*A*
O1*A*H1*OA*O1*W*	0.84(1)	1.90(1)	2.7227(19)	170(2)
O1*A*H2*OA*O2*W*	0.84(1)	1.79(1)	2.623(2)	173(2)
N1*A*H1*NA*Cl3*B* ^i^	0.98	2.43	3.3748(18)	163
N2*A*H2*NA*Cl2*B* ^ii^	0.98	2.64	3.4686(16)	142
N3*A*H3*NA*Cl3*B* ^i^	0.98	2.37	3.3403(15)	172
N4*A*H4*NA*Cl2*B*	0.98	2.48	3.4244(17)	163
O1*W*H1*O*1Cl4*B*	0.85(1)	2.33(1)	3.165(2)	171(3)
O1*W*H2*O*1Cl3*B* ^ii^	0.85(1)	2.59(1)	3.4029(18)	160(2)
O2*W*H2*O*2O3*W*	0.86(1)	1.92(1)	2.756(3)	165(3)
O3*W*H1*O*3O1*W* ^iii^	0.87(1)	2.02(2)	2.846(3)	158(3)
O3*W*H2*O*3Cl1*B* ^iii^	0.87(1)	2.52(1)	3.383(3)	174(4)

Symmetry codes: (i) 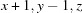; (ii) 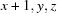; (iii) 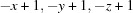.

**Table 2:** Experimental details

Crystal data	
Chemical formula	[CrCl(C_16_H_36_N_4_)(H_2_O)][ZnCl_4_]3H_2_O
*M*_r_	651.17
Crystal system, space group	Triclinic, *P* 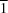
Temperature (K)	260
*a*, *b*, *c* ()	9.1010(18), 9.5830(19), 17.007(3)
, , ()	81.73(3), 75.80(3), 74.90(3)
*V* (^3^)	1383.2(6)
*Z*	2
Radiation type	Synchrotron, = 0.610
(mm^1^)	1.16
Crystal size (mm)	0.22 0.16 0.08

Data collection	
Diffractometer	ADSC Q210 CCD area detector
Absorption correction	Empirical (using intensity measurements) (*HKL3000sm SCALEAPCK*; Otwinowski Minor, 1997 ▸)
*T*_min_, *T*_max_	0.787, 0.917
No. of measured, independent and observed [*I* > 2(*I*)] reflections	14317, 7413, 7053
*R*_int_	0.013
(sin /)_max_ (^1^)	0.693

Refinement	
*R*[*F* ^2^ > 2(*F* ^2^)], *wR*(*F* ^2^), *S*	0.028, 0.080, 1.03
No. of reflections	7413
No. of parameters	310
No. of restraints	12
H-atom treatment	H atoms treated by a mixture of independent and constrained refinement
_max_, _min_ (e ^3^)	0.76, 0.56

Computer programs: *PAL ADSC Quantum-210 ADX* (Arvai Nielsen, 1983 ▸), *HKL3000sm* (Otwinowski Minor, 1997 ▸), *SHELXT2014/5* (Sheldrick, 2015*a* ▸), *SHELXL2014/7* (Sheldrick, 2015*b* ▸), *DIAMOND* (Putz Brandenburg, 2014 ▸) and *publCIF* (Westrip, 2010 ▸).

## supplementary crystallographic information

### Crystal data

**Table:**

[CrCl(C_16_H_38_N_4_O)(H_2_O)][ZnCl_4_]3H_2_O	*Z* = 2
*M**_r_* = 651.17	*F*(000) = 678
Triclinic, *P*1	*D*_x_ = 1.563 Mg m^−^^3^
*a* = 9.1010 (18) Å	Synchrotron radiation, λ = 0.610 Å
*b* = 9.5830 (19) Å	Cell parameters from 68409 reflections
*c* = 17.007 (3) Å	θ = 0.4–33.7°
α = 81.73 (3)°	µ = 1.16 mm^−^^1^
β = 75.80 (3)°	*T* = 260 K
γ = 74.90 (3)°	Plate, purple
*V* = 1383.2 (6) Å^3^	0.22 × 0.16 × 0.08 mm

### Data collection

**Table:**

ADSC Q210 CCD area detector diffractometer	7053 reflections with *I* > 2σ(*I*)
Radiation source: PLSII 2D bending magnet	*R*_int_ = 0.013
ω scan	θ_max_ = 25.0°, θ_min_ = 2.3°
Absorption correction: empirical (using intensity measurements) (*HKL3000sm SCALEAPCK*; Otwinowski & Minor, 1997)	*h* = −12→12
*T*_min_ = 0.787, *T*_max_ = 0.917	*k* = −13→13
14317 measured reflections	*l* = −23→23
7413 independent reflections	

### Refinement

**Table:**

Refinement on *F*^2^	12 restraints
Least-squares matrix: full	Hydrogen site location: difference Fourier map
*R*[*F*^2^ > 2σ(*F*^2^)] = 0.028	H atoms treated by a mixture of independent and constrained refinement
*wR*(*F*^2^) = 0.080	*w* = 1/[σ^2^(*F*_o_^2^) + (0.0439*P*)^2^ + 0.7948*P*] where *P* = (*F*_o_^2^ + 2*F*_c_^2^)/3
*S* = 1.03	(Δ/σ)_max_ = 0.001
7413 reflections	Δρ_max_ = 0.76 e Å^−^^3^
310 parameters	Δρ_min_ = −0.56 e Å^−^^3^

### Special details

**Table:**

Geometry. All e.s.d.'s (except the e.s.d. in the dihedral angle between two l.s. planes) are estimated using the full covariance matrix. The cell e.s.d.'s are taken into account individually in the estimation of e.s.d.'s in distances, angles and torsion angles; correlations between e.s.d.'s in cell parameters are only used when they are defined by crystal symmetry. An approximate (isotropic) treatment of cell e.s.d.'s is used for estimating e.s.d.'s involving l.s. planes.

### Fractional atomic coordinates and isotropic or equivalent isotropic displacement parameters (Å^2^)

**Table:**

	*x*	*y*	*z*	*U*_iso_*/*U*_eq_	
Cr1A	0.87300 (2)	0.33415 (2)	0.22043 (2)	0.01468 (5)	
O1A	0.69575 (12)	0.46877 (12)	0.28842 (7)	0.0251 (2)	
H1OA	0.697 (2)	0.5566 (12)	0.2814 (12)	0.030*	
H2OA	0.6213 (18)	0.456 (2)	0.3265 (9)	0.030*	
N1A	0.81211 (13)	0.15969 (13)	0.30221 (7)	0.0189 (2)	
H1NA	0.8881	0.0712	0.2837	0.023*	
N2A	1.05645 (13)	0.31721 (13)	0.28159 (7)	0.0203 (2)	
H2NA	1.0888	0.4090	0.2658	0.024*	
N3A	1.04622 (12)	0.20987 (12)	0.13663 (7)	0.01725 (19)	
H3NA	1.0404	0.1085	0.1514	0.021*	
N4A	0.70640 (12)	0.30026 (12)	0.15997 (7)	0.01734 (19)	
H4NA	0.6126	0.3769	0.1762	0.021*	
C1A	0.65887 (16)	0.14998 (17)	0.28964 (9)	0.0235 (3)	
H1A1	0.5768	0.2272	0.3153	0.028*	
H1A2	0.6365	0.0579	0.3143	0.028*	
C2A	0.80792 (17)	0.16149 (17)	0.39133 (8)	0.0240 (3)	
H2A	0.7315	0.2485	0.4116	0.029*	
C3A	0.7586 (2)	0.0277 (2)	0.44022 (11)	0.0397 (4)	
H3A1	0.6521	0.0331	0.4397	0.060*	
H3A2	0.7689	0.0244	0.4954	0.060*	
H3A3	0.8241	−0.0582	0.4162	0.060*	
C4A	0.96572 (18)	0.16400 (17)	0.40594 (9)	0.0265 (3)	
H4A1	1.0411	0.0803	0.3827	0.032*	
H4A2	0.9586	0.1505	0.4643	0.032*	
C5A	1.03257 (18)	0.29722 (17)	0.37339 (9)	0.0261 (3)	
C6A	0.9269 (2)	0.4363 (2)	0.40828 (10)	0.0368 (4)	
H6A1	0.9717	0.5170	0.3845	0.055*	
H6A2	0.9165	0.4277	0.4662	0.055*	
H6A3	0.8260	0.4517	0.3961	0.055*	
C7A	1.1889 (2)	0.2726 (2)	0.39878 (11)	0.0404 (4)	
H7A1	1.2530	0.1795	0.3840	0.061*	
H7A2	1.1702	0.2759	0.4566	0.061*	
H7A3	1.2411	0.3470	0.3716	0.061*	
C8A	1.18917 (16)	0.20584 (17)	0.24069 (9)	0.0252 (3)	
H8A1	1.1756	0.1097	0.2626	0.030*	
H8A2	1.2858	0.2164	0.2509	0.030*	
C9A	1.19721 (15)	0.22306 (16)	0.15068 (9)	0.0227 (3)	
H9A1	1.2163	0.3171	0.1280	0.027*	
H9A2	1.2820	0.1488	0.1242	0.027*	
C10A	1.03870 (15)	0.24351 (15)	0.04815 (8)	0.0200 (2)	
H10A	1.0335	0.3471	0.0335	0.024*	
C11A	1.18404 (18)	0.15683 (19)	−0.00605 (9)	0.0297 (3)	
H11A	1.2726	0.1933	−0.0057	0.045*	
H11B	1.1691	0.1664	−0.0607	0.045*	
H11C	1.2016	0.0564	0.0142	0.045*	
C12A	0.89375 (16)	0.20959 (17)	0.03353 (8)	0.0237 (3)	
H12A	0.8925	0.1108	0.0559	0.028*	
H12B	0.9051	0.2119	−0.0248	0.028*	
C13A	0.73394 (15)	0.30643 (16)	0.06770 (8)	0.0214 (2)	
C14A	0.71987 (19)	0.46457 (18)	0.03395 (10)	0.0304 (3)	
H14A	0.6183	0.5209	0.0569	0.046*	
H14B	0.7345	0.4711	−0.0242	0.046*	
H14C	0.7979	0.5012	0.0480	0.046*	
C15A	0.60817 (18)	0.2528 (2)	0.04261 (10)	0.0312 (3)	
H15A	0.6160	0.1523	0.0615	0.047*	
H15B	0.6232	0.2639	−0.0156	0.047*	
H15C	0.5069	0.3087	0.0663	0.047*	
C16A	0.66416 (16)	0.16285 (16)	0.19998 (8)	0.0225 (3)	
H16A	0.7403	0.0808	0.1754	0.027*	
H16B	0.5631	0.1610	0.1916	0.027*	
Zn1B	0.26864 (2)	0.74837 (2)	0.24042 (2)	0.02510 (6)	
Cl1A	0.93837 (4)	0.54067 (4)	0.15097 (2)	0.02727 (8)	
Cl1B	0.22710 (8)	0.75375 (7)	0.37777 (3)	0.05834 (16)	
Cl2B	0.33787 (5)	0.51602 (4)	0.20871 (3)	0.03437 (9)	
Cl3B	0.03474 (4)	0.86751 (4)	0.20538 (3)	0.03322 (9)	
Cl4B	0.45104 (6)	0.87103 (6)	0.17503 (4)	0.05119 (14)	
O1W	0.70053 (18)	0.75266 (16)	0.28427 (10)	0.0450 (3)	
H1O1	0.639 (2)	0.776 (3)	0.2521 (13)	0.054*	
H2O1	0.7866 (17)	0.766 (3)	0.2551 (13)	0.054*	
O2W	0.46628 (19)	0.4464 (2)	0.41394 (11)	0.0582 (4)	
H1O2	0.482 (4)	0.513 (2)	0.4363 (16)	0.070*	
H2O2	0.446 (4)	0.389 (3)	0.4566 (12)	0.070*	
O3W	0.4581 (3)	0.2496 (3)	0.54916 (14)	0.0858 (7)	
H1O3	0.389 (3)	0.247 (5)	0.5950 (13)	0.103*	
H2O3	0.540 (3)	0.254 (5)	0.565 (2)	0.103*	

### Atomic displacement parameters (Å^2^)

**Table:**

	*U*^11^	*U*^22^	*U*^33^	*U*^12^	*U*^13^	*U*^23^
Cr1A	0.01303 (9)	0.01372 (10)	0.01588 (9)	−0.00341 (7)	−0.00166 (7)	0.00118 (7)
O1A	0.0238 (5)	0.0196 (5)	0.0268 (5)	−0.0031 (4)	0.0030 (4)	−0.0036 (4)
N1A	0.0197 (5)	0.0179 (5)	0.0180 (5)	−0.0061 (4)	−0.0029 (4)	0.0026 (4)
N2A	0.0199 (5)	0.0213 (6)	0.0211 (5)	−0.0073 (4)	−0.0066 (4)	0.0018 (4)
N3A	0.0140 (4)	0.0163 (5)	0.0194 (5)	−0.0034 (4)	−0.0011 (4)	0.0002 (4)
N4A	0.0138 (4)	0.0179 (5)	0.0188 (5)	−0.0025 (4)	−0.0030 (4)	0.0003 (4)
C1A	0.0207 (6)	0.0261 (7)	0.0243 (6)	−0.0118 (5)	−0.0024 (5)	0.0033 (5)
C2A	0.0271 (6)	0.0260 (7)	0.0174 (6)	−0.0083 (5)	−0.0031 (5)	0.0041 (5)
C3A	0.0512 (10)	0.0427 (10)	0.0282 (8)	−0.0253 (9)	−0.0096 (7)	0.0156 (7)
C4A	0.0307 (7)	0.0277 (7)	0.0212 (6)	−0.0081 (6)	−0.0089 (5)	0.0054 (5)
C5A	0.0309 (7)	0.0294 (7)	0.0218 (6)	−0.0109 (6)	−0.0111 (5)	0.0018 (5)
C6A	0.0519 (10)	0.0344 (9)	0.0285 (7)	−0.0128 (8)	−0.0108 (7)	−0.0079 (6)
C7A	0.0419 (9)	0.0540 (11)	0.0353 (8)	−0.0216 (8)	−0.0229 (7)	0.0080 (8)
C8A	0.0169 (5)	0.0281 (7)	0.0297 (7)	−0.0023 (5)	−0.0081 (5)	0.0004 (5)
C9A	0.0129 (5)	0.0260 (7)	0.0270 (6)	−0.0039 (5)	−0.0023 (4)	−0.0003 (5)
C10A	0.0182 (5)	0.0206 (6)	0.0179 (5)	−0.0029 (5)	−0.0003 (4)	−0.0005 (4)
C11A	0.0228 (6)	0.0346 (8)	0.0259 (7)	−0.0015 (6)	0.0031 (5)	−0.0082 (6)
C12A	0.0208 (6)	0.0283 (7)	0.0213 (6)	−0.0039 (5)	−0.0033 (5)	−0.0059 (5)
C13A	0.0187 (5)	0.0254 (7)	0.0192 (6)	−0.0029 (5)	−0.0059 (4)	−0.0001 (5)
C14A	0.0292 (7)	0.0299 (8)	0.0279 (7)	−0.0032 (6)	−0.0087 (6)	0.0091 (6)
C15A	0.0236 (6)	0.0428 (9)	0.0302 (7)	−0.0063 (6)	−0.0121 (6)	−0.0044 (6)
C16A	0.0210 (6)	0.0235 (7)	0.0250 (6)	−0.0103 (5)	−0.0056 (5)	0.0016 (5)
Zn1B	0.02438 (9)	0.02053 (9)	0.02709 (9)	−0.00146 (6)	−0.00336 (6)	−0.00237 (6)
Cl1A	0.03019 (17)	0.02080 (16)	0.02835 (16)	−0.00789 (13)	−0.00262 (13)	0.00291 (12)
Cl1B	0.0683 (3)	0.0656 (4)	0.0287 (2)	0.0098 (3)	−0.0092 (2)	−0.0134 (2)
Cl2B	0.02746 (17)	0.02228 (18)	0.0507 (2)	−0.00162 (14)	−0.00473 (15)	−0.00905 (15)
Cl3B	0.02720 (17)	0.02074 (17)	0.0509 (2)	−0.00139 (13)	−0.01244 (15)	−0.00133 (15)
Cl4B	0.0334 (2)	0.0375 (3)	0.0755 (4)	−0.01276 (19)	−0.0026 (2)	0.0102 (2)
O1W	0.0402 (7)	0.0364 (7)	0.0533 (8)	−0.0088 (6)	0.0012 (6)	−0.0074 (6)
O2W	0.0380 (7)	0.0645 (11)	0.0563 (10)	−0.0082 (7)	0.0090 (7)	0.0051 (8)
O3W	0.0905 (17)	0.1024 (19)	0.0614 (13)	−0.0315 (15)	0.0022 (11)	−0.0132 (12)

### Geometric parameters (Å, º)

**Table:**

Cr1A—O1A	2.0082 (13)	C7A—H7A1	0.9600
Cr1A—N3A	2.0837 (14)	C7A—H7A2	0.9600
Cr1A—N1A	2.1147 (13)	C7A—H7A3	0.9600
Cr1A—N2A	2.1352 (12)	C8A—C9A	1.502 (2)
Cr1A—N4A	2.1399 (12)	C8A—H8A1	0.9700
Cr1A—Cl1A	2.2940 (8)	C8A—H8A2	0.9700
O1A—H1OA	0.835 (9)	C9A—H9A1	0.9700
O1A—H2OA	0.835 (9)	C9A—H9A2	0.9700
N1A—C1A	1.4896 (17)	C10A—C12A	1.5217 (19)
N1A—C2A	1.5093 (17)	C10A—C11A	1.529 (2)
N1A—H1NA	0.9800	C10A—H10A	0.9800
N2A—C8A	1.487 (2)	C11A—H11A	0.9600
N2A—C5A	1.5134 (18)	C11A—H11B	0.9600
N2A—H2NA	0.9800	C11A—H11C	0.9600
N3A—C9A	1.4905 (16)	C12A—C13A	1.533 (2)
N3A—C10A	1.5074 (17)	C12A—H12A	0.9700
N3A—H3NA	0.9800	C12A—H12B	0.9700
N4A—C16A	1.4909 (17)	C13A—C14A	1.526 (2)
N4A—C13A	1.5221 (17)	C13A—C15A	1.538 (2)
N4A—H4NA	0.9800	C14A—H14A	0.9600
C1A—C16A	1.502 (2)	C14A—H14B	0.9600
C1A—H1A1	0.9700	C14A—H14C	0.9600
C1A—H1A2	0.9700	C15A—H15A	0.9600
C2A—C4A	1.523 (2)	C15A—H15B	0.9600
C2A—C3A	1.532 (2)	C15A—H15C	0.9600
C2A—H2A	0.9800	C16A—H16A	0.9700
C3A—H3A1	0.9600	C16A—H16B	0.9700
C3A—H3A2	0.9600	Zn1B—Cl2B	2.2569 (7)
C3A—H3A3	0.9600	Zn1B—Cl4B	2.2603 (9)
C4A—C5A	1.531 (2)	Zn1B—Cl1B	2.2789 (7)
C4A—H4A1	0.9700	Zn1B—Cl3B	2.3131 (8)
C4A—H4A2	0.9700	O1W—H1O1	0.847 (9)
C5A—C6A	1.528 (3)	O1W—H2O1	0.848 (9)
C5A—C7A	1.537 (2)	O2W—H1O2	0.845 (10)
C6A—H6A1	0.9600	O2W—H2O2	0.859 (10)
C6A—H6A2	0.9600	O3W—H1O3	0.874 (10)
C6A—H6A3	0.9600	O3W—H2O3	0.870 (10)
			
O1A—Cr1A—N3A	172.43 (5)	H6A1—C6A—H6A2	109.5
O1A—Cr1A—N1A	88.19 (5)	C5A—C6A—H6A3	109.5
N3A—Cr1A—N1A	97.08 (5)	H6A1—C6A—H6A3	109.5
O1A—Cr1A—N2A	101.33 (5)	H6A2—C6A—H6A3	109.5
N3A—Cr1A—N2A	84.43 (5)	C5A—C7A—H7A1	109.5
N1A—Cr1A—N2A	87.01 (5)	C5A—C7A—H7A2	109.5
O1A—Cr1A—N4A	87.37 (5)	H7A1—C7A—H7A2	109.5
N3A—Cr1A—N4A	87.77 (5)	C5A—C7A—H7A3	109.5
N1A—Cr1A—N4A	84.11 (5)	H7A1—C7A—H7A3	109.5
N2A—Cr1A—N4A	167.37 (5)	H7A2—C7A—H7A3	109.5
O1A—Cr1A—Cl1A	85.74 (4)	N2A—C8A—C9A	109.92 (12)
N3A—Cr1A—Cl1A	89.71 (4)	N2A—C8A—H8A1	109.7
N1A—Cr1A—Cl1A	170.35 (3)	C9A—C8A—H8A1	109.7
N2A—Cr1A—Cl1A	86.83 (4)	N2A—C8A—H8A2	109.7
N4A—Cr1A—Cl1A	103.07 (4)	C9A—C8A—H8A2	109.7
Cr1A—O1A—H1OA	116.7 (14)	H8A1—C8A—H8A2	108.2
Cr1A—O1A—H2OA	133.8 (14)	N3A—C9A—C8A	108.68 (11)
H1OA—O1A—H2OA	109.3 (17)	N3A—C9A—H9A1	110.0
C1A—N1A—C2A	111.26 (11)	C8A—C9A—H9A1	110.0
C1A—N1A—Cr1A	105.43 (8)	N3A—C9A—H9A2	110.0
C2A—N1A—Cr1A	118.74 (9)	C8A—C9A—H9A2	110.0
C1A—N1A—H1NA	106.9	H9A1—C9A—H9A2	108.3
C2A—N1A—H1NA	106.9	N3A—C10A—C12A	110.54 (11)
Cr1A—N1A—H1NA	106.9	N3A—C10A—C11A	110.80 (12)
C8A—N2A—C5A	112.59 (12)	C12A—C10A—C11A	109.63 (12)
C8A—N2A—Cr1A	105.17 (9)	N3A—C10A—H10A	108.6
C5A—N2A—Cr1A	122.52 (9)	C12A—C10A—H10A	108.6
C8A—N2A—H2NA	105.0	C11A—C10A—H10A	108.6
C5A—N2A—H2NA	105.0	C10A—C11A—H11A	109.5
Cr1A—N2A—H2NA	105.0	C10A—C11A—H11B	109.5
C9A—N3A—C10A	111.61 (10)	H11A—C11A—H11B	109.5
C9A—N3A—Cr1A	105.81 (8)	C10A—C11A—H11C	109.5
C10A—N3A—Cr1A	117.28 (8)	H11A—C11A—H11C	109.5
C9A—N3A—H3NA	107.2	H11B—C11A—H11C	109.5
C10A—N3A—H3NA	107.2	C10A—C12A—C13A	118.61 (12)
Cr1A—N3A—H3NA	107.2	C10A—C12A—H12A	107.7
C16A—N4A—C13A	111.76 (11)	C13A—C12A—H12A	107.7
C16A—N4A—Cr1A	105.83 (8)	C10A—C12A—H12B	107.7
C13A—N4A—Cr1A	122.60 (8)	C13A—C12A—H12B	107.7
C16A—N4A—H4NA	105.1	H12A—C12A—H12B	107.1
C13A—N4A—H4NA	105.1	N4A—C13A—C14A	108.13 (12)
Cr1A—N4A—H4NA	105.1	N4A—C13A—C12A	109.83 (11)
N1A—C1A—C16A	109.24 (11)	C14A—C13A—C12A	112.37 (12)
N1A—C1A—H1A1	109.8	N4A—C13A—C15A	110.71 (11)
C16A—C1A—H1A1	109.8	C14A—C13A—C15A	107.32 (12)
N1A—C1A—H1A2	109.8	C12A—C13A—C15A	108.47 (12)
C16A—C1A—H1A2	109.8	C13A—C14A—H14A	109.5
H1A1—C1A—H1A2	108.3	C13A—C14A—H14B	109.5
N1A—C2A—C4A	112.13 (11)	H14A—C14A—H14B	109.5
N1A—C2A—C3A	110.63 (13)	C13A—C14A—H14C	109.5
C4A—C2A—C3A	108.18 (13)	H14A—C14A—H14C	109.5
N1A—C2A—H2A	108.6	H14B—C14A—H14C	109.5
C4A—C2A—H2A	108.6	C13A—C15A—H15A	109.5
C3A—C2A—H2A	108.6	C13A—C15A—H15B	109.5
C2A—C3A—H3A1	109.5	H15A—C15A—H15B	109.5
C2A—C3A—H3A2	109.5	C13A—C15A—H15C	109.5
H3A1—C3A—H3A2	109.5	H15A—C15A—H15C	109.5
C2A—C3A—H3A3	109.5	H15B—C15A—H15C	109.5
H3A1—C3A—H3A3	109.5	N4A—C16A—C1A	110.80 (11)
H3A2—C3A—H3A3	109.5	N4A—C16A—H16A	109.5
C2A—C4A—C5A	119.11 (12)	C1A—C16A—H16A	109.5
C2A—C4A—H4A1	107.5	N4A—C16A—H16B	109.5
C5A—C4A—H4A1	107.5	C1A—C16A—H16B	109.5
C2A—C4A—H4A2	107.5	H16A—C16A—H16B	108.1
C5A—C4A—H4A2	107.5	Cl2B—Zn1B—Cl4B	111.49 (3)
H4A1—C4A—H4A2	107.0	Cl2B—Zn1B—Cl1B	109.60 (4)
N2A—C5A—C6A	108.55 (13)	Cl4B—Zn1B—Cl1B	110.65 (4)
N2A—C5A—C4A	109.66 (12)	Cl2B—Zn1B—Cl3B	110.73 (3)
C6A—C5A—C4A	112.68 (14)	Cl4B—Zn1B—Cl3B	108.19 (3)
N2A—C5A—C7A	110.36 (13)	Cl1B—Zn1B—Cl3B	106.02 (4)
C6A—C5A—C7A	107.49 (14)	H1O1—O1W—H2O1	104.5 (18)
C4A—C5A—C7A	108.08 (13)	H1O2—O2W—H2O2	98.6 (19)
C5A—C6A—H6A1	109.5	H1O3—O3W—H2O3	102 (2)
C5A—C6A—H6A2	109.5		
			
C2A—N1A—C1A—C16A	174.65 (12)	Cr1A—N3A—C9A—C8A	44.64 (12)
Cr1A—N1A—C1A—C16A	44.67 (13)	N2A—C8A—C9A—N3A	−58.03 (15)
C1A—N1A—C2A—C4A	177.23 (12)	C9A—N3A—C10A—C12A	172.32 (11)
Cr1A—N1A—C2A—C4A	−60.17 (15)	Cr1A—N3A—C10A—C12A	−65.41 (13)
C1A—N1A—C2A—C3A	56.37 (16)	C9A—N3A—C10A—C11A	50.58 (15)
Cr1A—N1A—C2A—C3A	178.97 (11)	Cr1A—N3A—C10A—C11A	172.84 (9)
N1A—C2A—C4A—C5A	65.83 (18)	N3A—C10A—C12A—C13A	70.03 (16)
C3A—C2A—C4A—C5A	−171.90 (14)	C11A—C10A—C12A—C13A	−167.54 (13)
C8A—N2A—C5A—C6A	164.61 (12)	C16A—N4A—C13A—C14A	160.54 (11)
Cr1A—N2A—C5A—C6A	−68.42 (15)	Cr1A—N4A—C13A—C14A	−72.31 (13)
C8A—N2A—C5A—C4A	−71.90 (15)	C16A—N4A—C13A—C12A	−76.51 (13)
Cr1A—N2A—C5A—C4A	55.07 (15)	Cr1A—N4A—C13A—C12A	50.64 (14)
C8A—N2A—C5A—C7A	47.05 (17)	C16A—N4A—C13A—C15A	43.25 (15)
Cr1A—N2A—C5A—C7A	174.03 (11)	Cr1A—N4A—C13A—C15A	170.39 (10)
C2A—C4A—C5A—N2A	−61.30 (18)	C10A—C12A—C13A—N4A	−60.60 (16)
C2A—C4A—C5A—C6A	59.73 (18)	C10A—C12A—C13A—C14A	59.80 (16)
C2A—C4A—C5A—C7A	178.35 (14)	C10A—C12A—C13A—C15A	178.28 (12)
C5A—N2A—C8A—C9A	174.68 (11)	C13A—N4A—C16A—C1A	172.31 (11)
Cr1A—N2A—C8A—C9A	38.95 (12)	Cr1A—N4A—C16A—C1A	36.58 (12)
C10A—N3A—C9A—C8A	173.29 (11)	N1A—C1A—C16A—N4A	−56.58 (15)

### Hydrogen-bond geometry (Å, º)

**Table:**

*D*—H···*A*	*D*—H	H···*A*	*D*···*A*	*D*—H···*A*
O1*A*—H1*OA*···O1*W*	0.84 (1)	1.90 (1)	2.7227 (19)	170 (2)
O1*A*—H2*OA*···O2*W*	0.84 (1)	1.79 (1)	2.623 (2)	173 (2)
N1*A*—H1*NA*···Cl3*B*^i^	0.98	2.43	3.3748 (18)	163
N2*A*—H2*NA*···Cl2*B*^ii^	0.98	2.64	3.4686 (16)	142
N3*A*—H3*NA*···Cl3*B*^i^	0.98	2.37	3.3403 (15)	172
N4*A*—H4*NA*···Cl2*B*	0.98	2.48	3.4244 (17)	163
O1*W*—H1*O*1···Cl4*B*	0.85 (1)	2.33 (1)	3.165 (2)	171 (3)
O1*W*—H2*O*1···Cl3*B*^ii^	0.85 (1)	2.59 (1)	3.4029 (18)	160 (2)
O2*W*—H2*O*2···O3*W*	0.86 (1)	1.92 (1)	2.756 (3)	165 (3)
O3*W*—H1*O*3···O1*W*^iii^	0.87 (1)	2.02 (2)	2.846 (3)	158 (3)
O3*W*—H2*O*3···Cl1*B*^iii^	0.87 (1)	2.52 (1)	3.383 (3)	174 (4)

Symmetry codes: (i) *x*+1, *y*−1, *z*; (ii) *x*+1, *y*, *z*; (iii) −*x*+1, −*y*+1, −*z*+1.
